# Differential Efficacy of B-Ultrasound Combined with Molybdenum Target Detection Mode for Breast Cancer Staging and Correlation of Blood Flow Parameters with IGF-1 and IGF-2 Expression Level and Prognosis

**DOI:** 10.1155/2022/9198626

**Published:** 2022-06-24

**Authors:** Huiliao He, Guifan Zhang, Haixian Zhou, Chunyang Lin, Qun Xu, Ruixing Liu, Beibei Yu, Xiuping Zhou, Zhejin Wang, Zhihua Xu, Lejing Lin

**Affiliations:** ^1^Ultrasound Imaging Department of the Second Affiliated Hospital of Wenzhou Medical University, Wenzhou 325000, China; ^2^Anesthesiology Department, Wenzhou Integrated Traditional Chinese and Western Medicine Hospital, Wenzhou 325000, China; ^3^Obstetrics and Gynecology Department, Wenzhou Integrated Traditional Chinese and Western Medicine Hospital, Wenzhou 325000, China; ^4^Department of General Surgery, Wenzhou Central Hospital, The Second Affiliated Hospital of Shanghai University, Wenzhou 325000, China

## Abstract

The study investigates the diagnostic efficacy of ultrasound combined with the molybdenum target mode in breast cancer staging and the relationship between blood flow parameters and the expression of insulin-like growth factor 1 (IGF-1) and factor 2 (IGF-2) and prognosis. A total of 96 patients admitted to hospital from January 2020 to January 2021 are included in the breast cancer group, and 58 patients admitted to our hospital during the same period are included in the control group, who are diagnosed with benign breast lesions. All patients receive clinicopathological diagnosis, ultrasound detection, and X-ray molybdenum detection. Ultrasound detection, molybdenum target detection, ultrasound combined with the molybdenum target detection mode, and clinicopathological diagnosis results are compared. B-ultrasound combined with the molybdenum target detection mode has high efficiency in diagnosing breast cancer and differentiating pathological stages. Besides, blood flow parameters of patients are closely related to IGF-1 and IGF-2, and IGF-1 and IGF-2 expressions are closely related to the prognosis of patients. Subsequent diagnosis of the disease degree of breast cancer patients can be carried out by ultrasound combined with the molybdenum target detection mode. In addition, the expression of IGF-1 and IGF-2 in patients can be monitored to improve the clinical diagnosis and treatment plan to improve the prognosis of patients, which has a high clinical application value and is worth promoting.

## 1. Introduction

Breast cancer is a common type of malignant tumor disease in the female population, whose incidence is affected by a variety of factors and mechanisms. In recent years, clinical data show that the population of breast cancer patients has been increasing year by year and developing in a younger age, with poor prognosis and increasing mortality [1, 2]. The early symptoms of breast cancer are not obvious; when obvious signs appear, the patient has progressed to the middle and advanced stages, the optimal treatment time is missed, and the radical surgical treatment plan and radiotherapy and chemotherapy plan are difficult to completely cure the cancer, resulting in poor prognosis of patients [3]. Therefore, early assessment of breast cancer and accurate judgment of its disease development are of great significance for the formulation and improvement of follow-up clinical diagnosis and treatment plans and the realization of effective targeted treatment. More than usual, clinicals take X-ray mammography breast photography in breast cancer diagnosis; its main detection mechanism is around the edges of the patients with breast tumor, and according to its shape and glandular structure, microcalcifications are observed, and complete the clinical diagnosis, but the sample in the detection of breast lesions and the nature of the lesion in the identification of low accuracy lead to its clinical applications that have limitations [4, 5]. In recent years, the diagnostic value of ultrasound in breast cancer has been widely concerned. The ultrasound detection method can measure blood flow parameters at the lesions of patients and take this as an important indicator to evaluate the lesions of patients, providing objective reference for disease diagnosis and evaluation [6]. It should be noted that the pathogenesis of breast cancer is still unclear, and studies have shown that insulin-like growth factor plays an extremely important role in cell growth, differentiation, proliferation, migration, and apoptosis [7]. IGF-1 and IGF-2 mainly promote mitosis, regulate the synthesis and secretion of hormones in vivo, and also affect cell chemotaxis, immunity, and migration [8]. Related studies have shown that IGF-1 and IGF-2 are highly expressed in a variety of malignant tumors, suggesting that they are closely related to the occurrence and development of breast cancer. This study aims to explore the efficacy of ultrasound combined with the molybdenum target detection mode in clinical diagnosis and evaluation of the development of breast cancer and to perform IGF-1 and IGF-2 detection for patients with different degrees of disease.

A total of 96 patients admitted to our hospital for gynecological examination and diagnosed with breast cancer from January 2020 to January 2021 are included in the breast cancer group, and 58 patients with benign breast lesions admitted to our hospital during the same period are included in the control group. All patients are female, and the age range of patients in the breast cancer group is 28–68 years old. The mean age is (44.64 ± 4.27) years, the course of disease is 1–5 years, and the mean course of disease is (2.86 ± 0.83) years. The patients in the control group ranged in age from 30 to 66 years, with a mean age of (45.03 ± 4.36) years, and a mean disease course of (2.93 ± 0.94) years from 1 to 6 years. There are no significant statistical differences in baseline data such as age and disease course between the two groups, which confirmed that the comparison between groups is scientific and reasonable (all *P* > 0.05). Inclusion criteria include the following: all patients underwent pathological examination and confirmed the disease type, the patient is less than 70 years old, and the expected survival is more than 6 months, the disease stages of breast cancer patients are stages I–IV, and patients with clear consciousness and high clinical cooperation can cooperate with this study and related follow-up investigation until the end of the study. Exclusion criteria include the following: patients complicated with other benign and malignant tumor diseases, patients with severe heart, lung, liver, and kidney diseases, before participating in the study, the patients had received other treatment plans that might affect the results of the study, no blood flow signal is detected in the lesions of patients, and patients with contrast agent allergy.

The remainder of this study is organized as follows.  Section 2 presents the statistical method.  Section 3 provides the experimental result and Section 4 illustrates data analysis and result discussion. Finally, the conclusions of this study and some future recommendations are given in Section 5.

## 2. The Statistical Methods

MXR-550 digital molybdenum target mammary camera (Shenzhen Micron Electronics Co., Ltd., Guangdong) is used for detection. Mammary glands of the subjects are photographed, and both head and tail and internal and external oblique mammary glands are taken. The results of imaging examination are reviewed by 2 doctors with more than 3 years of working experience, and the size, number, shape, density, and edge of lesions are analyzed.

HY-K20 color Doppler ultrasound instrument (produced by Shanghai Huanxi Medical Instrument Co., Ltd.) is used, and the frequency of frequency conversion probe of the instrument is set to 5–12 MHz. Before detection, the patient is guided to keep supine position and expose both breasts. The echo, boundary, morphology, and whether there is microcalcification in breast lesions are recorded, and the blood flow imaging technology in the Doppler ultrasound instrument is used to check the distribution and morphology of blood flow in patients' lesions. No blood flow signal in and around 0.5 cm is considered as no blood flow. There are blood flow signals in and around the mass in the range of 0.5 cm, and at most 1-2 blood flow is small. Blood flow signals are observed in and around the mass in the range of 0.5 cm. The pulsatility index (PI), resistance index (RI), and peak flow rate (*V*_max_) are calculated.

5 mL fasting venous blood is taken from all the subjects in the morning, and the blood samples are centrifuged at 3500 r/min with a centrifugation radius of 10 cm and a centrifugation time of 15 min. After the operation, the supernatant is taken and IGF-1 and IGF-2 levels are detected by ELISA. The kits are purchased from American R&D company. Complete the corresponding operation in strict accordance with the instruction.

SPSS 26.0 software is used, counting data are described by (*n*,%), the *x*^2^ test is used for intergroup comparison, the Kappa consistency test is used for consistency comparison, and Pearson' and Spearman' correlation coefficients are used for relationship analysis. *P* < 0.05 is considered to be statistically significant.

## 3. The Experimental Results

### 3.1. Comparison between Molybdenum Target Detection and Clinicopathological Diagnosis

Pathological biopsy confirms that there are 58 benign tumor patients (stage 0), 21 breast cancer patients (stage I), 33 stage II, 22 stage III, and 20 stage IV. The Kappa consistency test shows that there is a good consistency between molybdenum target detection and pathological results (Kappa value = 0.675, *P* = 0.000).  Table 1 provides the comparison of molybdenum target detection and clinicopathological diagnosis results.

### 3.2. Comparison of Ultrasound Detection Mode and Clinicopathological Diagnosis Results

Pathological biopsy confirms that there are 58 benign tumor patients (stage 0), 21 breast cancer patients (stage I), 33 stage II, 22 stage III, and 20 stage IV. The Kappa consistency test shows that there is a good consistency between ultrasonic detection and pathological results (Kappa value = 0.778, *P* = 0.000), which is higher than ultrasonic detection.  Table 2 provides the comparison of the ultrasonic detection mode and clinicopathological diagnosis results.

### 3.3. Comparison of Ultrasound Combined with Molybdenum Target Detection Mode and Clinicopathological Diagnosis Results

Pathological biopsy confirms that there are 58 benign tumor patients (stage 0), 21 breast cancer patients (stage I), 33 stage II, 22 stage III, and 20 stage IV. The Kappa consistency test shows that the consistency between molybdenum target test and pathological results is higher than that of the ultrasonic test, and the consistency of the combined test is higher than that of the ultrasonic and molybdenum target single test (Kappa value = 0.907, *P* = 0.000).  Table 3 provides the comparison of ultrasound combined with the molybdenum target detection mode and clinicopathological diagnosis results.

### 3.4. Blood Flow Parameters of the Two Groups Are Compared

Blood flow parameters including PI, RI, and *V*_max_ in the breast cancer group increased significantly than those in the control group (all *P* < 0.05).  Table 4 provides the comparison of blood flow parameters.

### 3.5. IGF-1 and IGF-2 Expressions Are Compared in Each Group

According to the clinicopathological diagnosis results, a subgroup is established for the breast cancer group, in which patients with stages I-II are included in the stages I-II group (*n* = 54), and patients with stages III-IV are included in the stages III-IV group (*n* = 42). The detection results show that IGF-1 and IGF-2 expressions are lower in the benign group than in the breast cancer patients. With the increase of tumor stage, the expressions of IGF-1 and IGF-2 show an upward trend.  Table 5 provides the comparison of IGF-1 and IGF-2 expression in each group.

### 3.6. Compare the 12-Month Prognosis of Patients in Different Groups of Breast Cancer

A 12-month follow-up survey is conducted for all breast cancer patients in this study, which shows that the recurrence, metastasis, and mortality of the stages III-IV breast cancer group increases significantly than that of the stages I-II breast cancer group after 12 months (*P* < 0.05).  Table 6 provides the prognosis of breast cancer patients in different groups at 12 months.

### 3.7. Correlation of IGF-1 and IGF-2 Expressions with Blood Flow Parameters and Prognosis in Breast Cancer Patients

**Figure 1:**
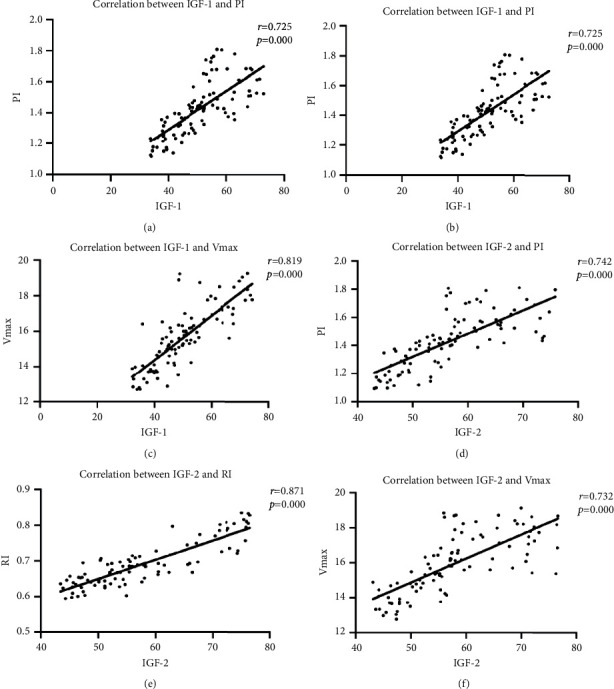
Correlation of IGF-1 and IGF-2 expression. (a) Correlation between IGF-1 and PI. (b) Correlation between IGF-1 and RI. (c) Correlation between IGF-1 and *V*_max_. (d) Correlation between IGF-2 and PI. (e) Correlation between IGF-2 and RI. (f) Correlation between IGF-2 and *V*_max_.

Pearson correlation coefficient is used to analyze the correlation between IGF-1 expression and blood flow parameters PI, RI, and *V*_max_ in breast cancer patients, showing positive correlation (all *P* < 0.05). Spearman correlation coefficient is used to analyze IGF-1 in breast cancer patients. IGF-2 expression and prognosis showed positive correlation (all *P* < 0.05).  Figure 1 shows the correlation of IGF-1 and IGF-2 expression.  Table 7 provides the correlation between IGF-1, IGF-2, and prognosis of breast cancer patients.

## 4. Data Analysis and Result Discussion

Clinical data show that the incidence of breast cancer in women is increasing day by day, and its pathogenesis is not clear and early symptoms are not obvious, resulting in low clinical diagnosis and treatment efficiency. Therefore, strengthening the early accurate diagnosis of breast cancer, patients timely receive symptomatic treatment of secondary prevention and treatment measures, in order to reduce the mortality of breast cancer, and improving the quality of life of patients is very important.

Clinical detection and diagnosis for breast cancer at the present stage opened imaging techniques, including X-ray mammography. It is a clinical breast examination technology commonly used at present; the technology is mainly around the patients with tumor location and its morphology, although mammography detected calcified lesions such as signs with high sensitivity. However, for some dense breast lesions, due to the influence of technology, the penetration of the imaging results is low, and some malignant breast tumor lesions in the shape of small nodules in patients are difficult to be effectively detected by X-ray molybdenum target.

Ultrasound, as an important product of the rising level of medical technology, has generally improved its clinical application rate. Compared with molybdenum target radiography technology, ultrasound can clearly observe the blood flow distribution and related blood flow parameters of patients. In addition, studies have pointed out that the complication and progression of breast cancer are closely related to new angiogenesis. Blood vessels induced by breast malignant tumor cells can be effectively evaluated by ultrasound. With the continuous development of imaging technology, ultrasound is highly sensitive to low blood flow and tiny new blood vessels in breast mass. Other scholars pointed out that in the most benign tumor, blood flow signal is less than normal blood vessels form and complete with wall structure. The blood flow signals in malignant tumor are more; the tumor tissue grew more quickly, causing vascular structures in chaos, pipe diameter, and the lack of regularity. So, malignant tumor blood flow parameters such as RI indexes are highly expressed. Therefore, many scholars have proposed that this detection can be used as a supplement to molybdenum target examination to make up for its deficiency in dense breast lesions and other aspects, thus helping to improve the diagnostic accuracy. This study, according to the results of ultrasonic testing compared with clinical pathology test results, prompts that joint detection can further help clinician's tumor staging in patients with breast cancer. It provides reliable basis for the establishment of disease treatment.

In addition, in order to further analyze the influencing mechanism of the occurrence and development of breast cancer disease, IGF-1 and IGF-2 are detected in this study for patients with benign lesions and breast cancer of different stages. It is found that all indicators in patients with benign lesions are of low expression. The expression levels of IGF-1 and IGF-2 are increased (both *P* < 0.05), suggesting that the changes of these two indicators may affect the severity and prognosis of breast cancer patients. In this study, correlation coefficient analysis shows that IGF-1 and IGF-2 expressions are positively correlated with blood flow parameters PI, RI, and *V*_max_ in breast cancer patients (all *P* < 0.05). The expression of IGF-1 and IGF-2 is positively correlated with the prognosis of patients with breast cancer (*P* < 0.05), which further confirmed that the expression of IGF-1 and IGF-2 had certain influence on blood flow parameters, which could be found by ultrasound examination, and affected the prognosis of patients with breast cancer. It may be that IGF-1 can stimulate the estrogen activity of breast cancer patients, improve the estrogen level, and then induce the proliferation and migration of breast cancer cells. IGF-2 can directly act on cells, promote tumor angiogenesis, inhibit tumor cell apoptosis, and lead to cell proliferation and division, thereby affecting the prognosis of patients.

## 5. Conclusion

In conclusion, b-ultrasound combined with molybdenum target detection has high efficiency in the diagnosis of breast cancer and differential pathological stages, suggesting that combined diagnosis can improve the clinical diagnosis efficiency. Breast cancer patients with disease and illness development situation may be affected by the IGF-1 and IGF-2 expression. In turn, it has influence on patient's prognosis, degree of follow-up for breast cancer patients diagnosed by ultrasonography combined with the mammography detection mode. For patients with IGF-1 and IGF-2 expression monitoring, we should improve the clinical diagnosis and treatment schemes to improve the prognosis of patients. It has high clinical application value and is worth popularizing.

This study also has some limitations, such as limited sample size selection and short follow-up investigation time. In the follow-up, the number and scope of sample size should be expanded and the follow-up investigation time should be extended to further analyze the pathogenesis of breast cancer, so as to realize early diagnosis and reasonable basis for clinical improvement of effective diagnosis and treatment plans.

## Figures and Tables

**Table 1:** Comparison of molybdenum target detection and clinicopathological diagnosis results.

Molybdenum target detection	Pathological diagnosis					Combined
	0 phase	I phase	II phase	III phase	IV phase	
0 phase	52	4	0	0	0	56
I phase	6	14	5	0	0	25
II phase	0	3	24	7	0	34
III phase	0	0	4	11	5	20
IV phase	0	0	0	4	15	19
Combined	58	21	33	22	20	154

**Table 2:** Comparison of the ultrasonic detection mode and clinicopathological diagnosis results.

Ultrasonic testing	Pathological diagnosis					Combined
	0 phase	I phase	II phase	III phase	IV phase	
0 phase	53	3	0	0	0	56
I phase	5	17	5	0	0	27
II phase	0	1	27	5	0	33
III phase	0	0	1	14	3	18
IV phase	0	0	0	3	17	20
Combined	58	21	33	22	20	154

**Table 3:** Comparison of ultrasound combined with the molybdenum target detection mode and clinicopathological diagnosis results.

The joint detection	Pathological diagnosis					Combined
	0 phase	I phase	II phase	III phase	IV phase	
0 phase	56	1	0	0	0	57
I phase	2	20	4	0	0	26
II phase	0	0	29	0	0	29
III phase	0	0	0	20	2	22
IV phase	0	0	0	2	18	20
Combined	58	21	33	22	20	154

**Table 4:** Comparison of blood flow parameters.

Group	PI	RI	*V*_max_ (cm/s)
The control group (*n* = 58)	1.19 ± 0.17	0.53 ± 0.06	14.27 ± 1.62
Breast cancer group (*n* = 96)	1.58 ± 0.23	0.75 ± 0.09	17.25 ± 2.03
*t*	−11.192	−16.520	−9.497
*P*	<0.001	<0.001	<0.001

**Table 5:** Comparison of IGF-1 and IGF-2 expression in each group.

Group	IGF-1 (*μ*g/L)	IGF-2 (*μ*g/L)
Benign group (*n* = 58)	27.37 ± 4.48	28.68 ± 4.72
Breast cancer stages I-II group (*n* = 54)	37.93 ± 5.82	48.36 ± 5.42
Breast cancer stages III-IV group (*n* = 42)	68.26 ± 6.15	70.61 ± 6.47
*F*	−10.803	−20.529
*P*	<0.001	<0.001

**Table 6:** Prognosis of breast cancer patients in different groups at 12 months (*n*, %).

Group	Recurrence/metastasis	Death
Breast cancer stages I-II group (*n* = 54)	16 (29.63)	6 (11.11)
Breast cancer stages III-IV group (*n* = 42)	22 (40.74)	15 (27.78)
*x* ^2^	5.113	8.368
*P*	0.024	0.004

**Table 7:** Correlation between IGF-1, IGF-2, and prognosis of breast cancer patients.

	Incidence of adverse outcome	
	*rs*	*P*
IGF-1	0.735	<0.001
IGF-2	0.782	<0.001

## Data Availability

The data used to support the findings of this study are available from the corresponding author upon request.
